# Plasma gelsolin is associated with hip BMD in Chinese postmenopausal women

**DOI:** 10.1371/journal.pone.0197732

**Published:** 2018-05-22

**Authors:** Wen-Yu Wang, Bing Ge, Ju Shi, Xu Zhou, Long-Fei Wu, Chang-Hua Tang, Dong-Cheng Zhu, Hong Zhu, Xing-Bo Mo, Yong-Hong Zhang, Fei-Yan Deng, Shu-Feng Lei

**Affiliations:** 1 Center for Genetic Epidemiology and Genomics, School of Public Health, Medical College of Soochow University, Suzhou, Jiangsu, P. R. China; 2 Jiangsu Key Laboratory of Preventive and Translational Medicine for Geriatric Diseases, Soochow University, Suzhou, Jiangsu, P. R. China; 3 Department of Orthopedics, Sihong People's Hospital, Suqian, Jiangsu, P. R. China; Universite de Liege, BELGIUM

## Abstract

Gelsolin (GSN) protein, expressed in circulating monocytes, was previously reported to be associated with osteoporosis in both Chinese and Caucasian women. This study aims to test if plasma GSN protein level is associated with hip bone mineral density (BMD) in Chinese population. Based on two study Groups containing 6,308 old Chinese, we adopted extreme sampling scheme and selected 3 independent samples (Subgroups 1–3) for discovery, replication, and validation purposes. We tested plasma GSN concentration, and analyzed whether plasma GSN level differs between subjects with extremely low vs. high hip BMD. In Group 1 (N = 1,860), the plasma GSN level increased in the female with low BMD, which was discovered in the Subgroup 1 (N = 42, p = 0.093) and replicated in the Subgroup 2 (N = 39, p = 0.095). With more extreme sampling for the Subgroup 3 from the Group 2 (N = 4,448), the difference of plasma GSN level in the female with low BMD vs. high BMD is more significant (N = 45, p = 0.037). After the subjects were pooled from Subgroups 2 and 3, the difference in plasma GSN between low and high BMD subjects became even more significant (p = 0.016). The plasma GSN level was negatively correlated with total hip BMD (r = -0.26, p = 0.033). We concluded that plasma GSN was associated with hip BMD in Chinese postmenopausal women and plasma GSN might be a potential risk biomarker for osteoporosis.

## Introduction

Osteoporosis (OP) is a chronic bone disease, which is an increasingly serious public health problem around the world. Its main characteristics are low bone mineral density (BMD) and destruction of micro architecture, which induce the sharp increased risk of osteoporotic fractures (OF) mostly happening at hip and spine [1]. Hip osteoporotic fracture is the most devastating consequence with high mortality and disability [2]. It has been reported that about 2 million men and 8 million women have been suffering from OP in USA, meanwhile there were estimated 34 million people suffering from osteopenia [3]. In Europe, approximately 5.5 million males and 22 million females have been diagnosed as OP [4]. In China, the prevalence has been increasing from 14.94% before 2008 to 27.96% in 2012–2015, which brings a huge social and economic burden[5].

Gelsolin (GSN) is a founding protein of the gelsolin superfamily[6], and has various functions in tissues and organs of mammals and non-mammals[7]. It plays an important role in severing assembled actin filaments, capping the fast growing plus ends and encouraging the growth of actin filaments in human under the regulation of calcium ions and pH, which can stimulate GSN binding to actin[8]. Recent studies showed that GSN concentration was related to many diseases, such as breast cancer, oral carcinoma, multiple sclerosis and rheumatoid arthritis [9–12]. GSN is also related to function of osteoclast, e.g., the mice lacking GSN expression would lead to the decrease of bone resorption and the increase of bone mass and strength[13].

A clinical proteomics study showed that GSN protein expression level in circulating monocytes (CMCs, osteoclast precursors) differs significantly in Chinese premenopausal females with extremely discordant BMD[14]. Later on, a CMC proteome-based multi-disciplinary and integrative study in premenopausal Caucasian women showed that GSN is significant for BMD in Caucasians as well[15]. These results, taken together, highlighted the high importance of GSN for BMD and bone metabolism. Moreover, GSN has potential capacity of being secreted out from CMCs. It was unknown whether plasma GSN is associated with BMD and could be a potential biomarker for OP.

To address the above question, we adopted an extreme sampling design and tested whether plasma GSN is correlated with human BMD and bone turnover markers in two independent Chinese populations. Our results showed the plasma GSN concentration was negatively correlated with total hip BMD in Chinese postmenopausal women, suggesting that plasma GSN is a potential osteoporosis biomarker.

## Materials and methods

### Samples

**Fig 1:**
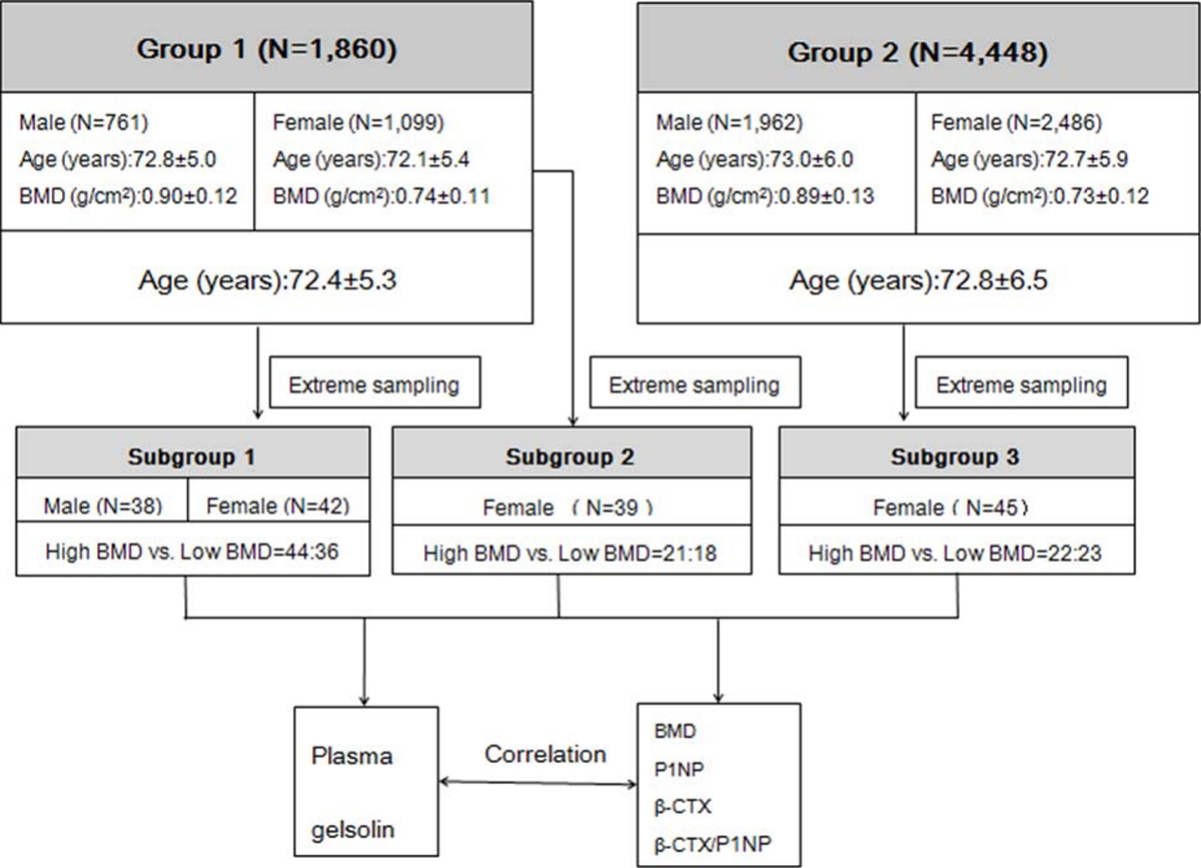
An overview of the flow chart for this study.

The flow chart of the study, including the sampling scheme, was presented in **Fig 1**. We first recruited two study Groups (N = 6,308) composed of old Chinese(>65 year). The Group 1 consisted of 1,860 subjects (Male: 761, aged 72.8±5.0; Female: 1,099, aged 72.1±5.4 years),and the Group 2 consisted of 4,448 subjects (Male: 1,962, aged 73.0±6.0; Female: 2,486, aged 72.7±5.9 years). All the females were postmenopausal women. The two groups were selected from an ongoing Osteoporosis Prevention Project (OPP), which was aimed at comprehensively discovering OP biomarkers, building early warning models, and developing early intervention of OP. Specific information, such as age, height, weight, disease history and medical history, etc., was collected by questionnaire.

Based on the Groups 1 and 2, an extreme sampling method was used to generate three independent samples (Subgroups 1–3) with extremely high and low BMD. We sorted the total hip BMD from highest to lowest, then selected subjects from the top or bottom 10% of the BMD distribution. The Subgroups 1 and 2, which were independent, generated from Group 1, were used for discovery and replication, respectively. The Subgroup 3 was generated from Group 2 and used for validation. The basic characteristicsof the three Subgroups were presented in **Fig 1**.

To minimize potential confounding effects on bone mass, strict exclusion criteria were adopted. These excusing diseases and conditions included chronic disorders involving vital organs (e.g., heart, lung, liver, kidney, and brain), serious metabolic diseases (e.g., diabetes, hypo- or hyperparathyroidism, and hyperthyroidism), other skeletal diseases (e.g., Paget disease, and rheumatoid arthritis), chronic use of drugs affecting bone metabolism (e.g., corticosteroid therapy and anticonvulsant drugs).

This study was approved by Institutional Research Ethic Board at the Soochow University. All subjects came from southeast China in Suzhou City, Jiangsu province. Before entering the study, every volunteer had provided written informed consents.

### BMD measurement

Total hip BMD value (g/cm^2^) was combined by the value of the femoral neck, trochanter and intertrochanter measured with dual-energy X-ray absorptiometry (DXA) with Hologic densitometers (Hologic Inc., Waltham, MA, USA), and all DXA measurements were performed by the trained operators. The precision of BMD, expressed as the root-mean-square percent coefficient of variation (RMS-CV), was evaluated by randomly testing thirty subjects for three times. The RMS-CV for hip BMD was 2.49%.

### Measurements of GSN, bone turnover markers, and metabolism indexes

Venous blood was drawn from each subject at 7:00–9:00 in the morning and the plasma was extracted from anti-aggregation whole blood and kept it in -80°C deep freezer for later usage. The concentrations of plasma glucose, total cholesterol and triglycerides were measured by an auto-analyzer. Plasma GSN levels were determined by enzyme-linked immuno sorbent assay (ELISA). Procollagen type I amino-terminal propeptide (P1NP) and β-isomerization of the C-terminal telopeptide of type I collagen (CTX) are two commonly used biomarkers in the plasma for measuring the rate of bone turnover. The concentrations of β-CTX and P1NP were determined by ELISA (FeikangBiotec, Guangzhou, China).

### Data analysis

All data were shown as mean±SD. We adopted Student’s t-test, analysis of covariance, Pearson’s correlation analysis, and partial correlation to analyze the relationship of plasma GSN concentration with other metabolic indexes. The SAS 9.2 software or SPSS 19.0 software was used for all statistical analyses. P<0.05 and P<0.1 were defined to be statistically significant and suggestive, respectively.

## Results

### Basic characteristics of the studied Subgroups

The subjects’ basic characteristics and metabolic indexes were summarized in **Table 1**. As expected, total hip BMD between extremely high and low subjects differ significantly in the three Subgroups. The markers of bone turnover (P1NP, β-CTX and β-CTX/P1NP) presented significant differences in some situations, e.g., P1NP in the Subgroup 3, β-CTX in the Subgroup 1 Male, and β-CTX/P1NP in the Subgroup 1 Total. Among the other three metabolic indexes (blood glucose, total cholesterol, triglyceride), only blood glucose showed difference in some situations, e.g., in the Subgroup 2. Total cholesterol and triglycerides had no significant differences in any of the three Subgroups.

**Table 1:** Basic characteristics of the three independent extreme samples (Subgroups 1–3).

	Subgroup 1						Subgroup 2 (All females)		Subgroup 3 (All females)	
	Male		Female		Total					
	High BMD	Low BMD	High BMD	Low BMD	High BMD	Low BMD	High BMD	Low BMD	High BMD	Low BMD
N	20	18	24	18	44	36	21	18	22	23
Age (year)	70.0±3.3	70.9±2.8	69.0±2.4	70.8±2.4*	70.8±2.8	69.4±2.6*	69.0±2.4	70.8±2.4*	68.0±3.3	70.0±2.5*
Weight (kg)	74.3±7.4	60.9±9.5*	64.5±6.6	49.8±5.2*	68.9±8.4	55.2±9.3*	64.4±6.9	49.8±5.2*	70.7±8.3	46.8±6.6*
Height (cm)	164.2±6.2	163.9±7.03	158.2±8.1	151.1±5.0*	160.8±7.8	157.4±8.8	158.7±8.1	151.1±5.0*	157.3±3.6	151.0±4.7*
BMI (kg/m2)	27.6±2.3	22.6±3.3*	25.9±3.0	21.8±2.2*	26.7±2.8	22.2±2.8*	25.7±3.1	21.8±2.2*	28.5±2.86	20.6±3.3*
BMD (g/cm2)	1.14±0.06	0.71±0.02*	0.98±0.05	0.54±0.05*	1.05±0.09	0.63±0.09*	0.98±0.04	0.54±0.05*	1.02±0.07	0.51±0.04*
P1NP (ng/ml)	39.90±15.67	34.73±7.71	38.95±12.11	51.18±22.23*	39.4±13.6	42.9±18.3	39.31±12.07	51.18±22.23*	41.05±15.73	53.48±17.00*
β-CTx (ng/ml)	0.14±0.10	0.30±0.22*	0.22±0.12	0.20±0.11	0.23±0.17	0.35±0.19*	0.22±0.13	0.20±0.11	0.24±0.23	0.42±0.39
β-CTx/P1NP (10^−2^)	0.34±0.20	0.86±0.52*	0.61±0.48	0.59±0.48	0.63±0.55	0.97±0.77*	0.64±0.50	0.59±0.48	0.52±0.41	0.65±0.60
Blood glucose (mmol/L)	5.80±1.42	5.45±0.65	6.32±1.52	5.35±0.37*	6.08±1.48	5.40±0.52*	6.27±0.59	5.35±0.37*	6.26±1.32	6.68±4.04
Total cholesterol (mmol/L)	3.62±0.68	3.81±0.65	3.98±0.47	3.91±0.78	3.81±0.59	3.86±0.71	3.99±0.40	3.91±0.78	5.24±0.93	5.04±0.87
Triglyceride (mmol/L)	1.56±0.91	1.41±0.89	1.62±0.72	1.42±0.44	1.59±0.80	1.41±0.69	1.55±0.69	1.42±0.44	1.60±0.97	1.35±0.77
Gelsolin (ng/ml)	237.14±88.17	247.91±59.46	228.44±85.37	260.43±92.42	232.39±78.86	253.99±83.28	169.17±59.10	195.13±50.87	199.16±75.03	230.08±81.89*

Data are presented as mean ± SD.

*P < 0.05 for comparison of high BMD vs. low BMD, and comparisons are not controlled for any variables.

BMD, bone mineral density; P1NP, procollagen type I amino-terminal propeptide; β-CTX, β-isomerization of the C-terminal telopeptide of type I collagen.

The difference of plasma GSN between low vs. high BMD females.

**Fig 2:**
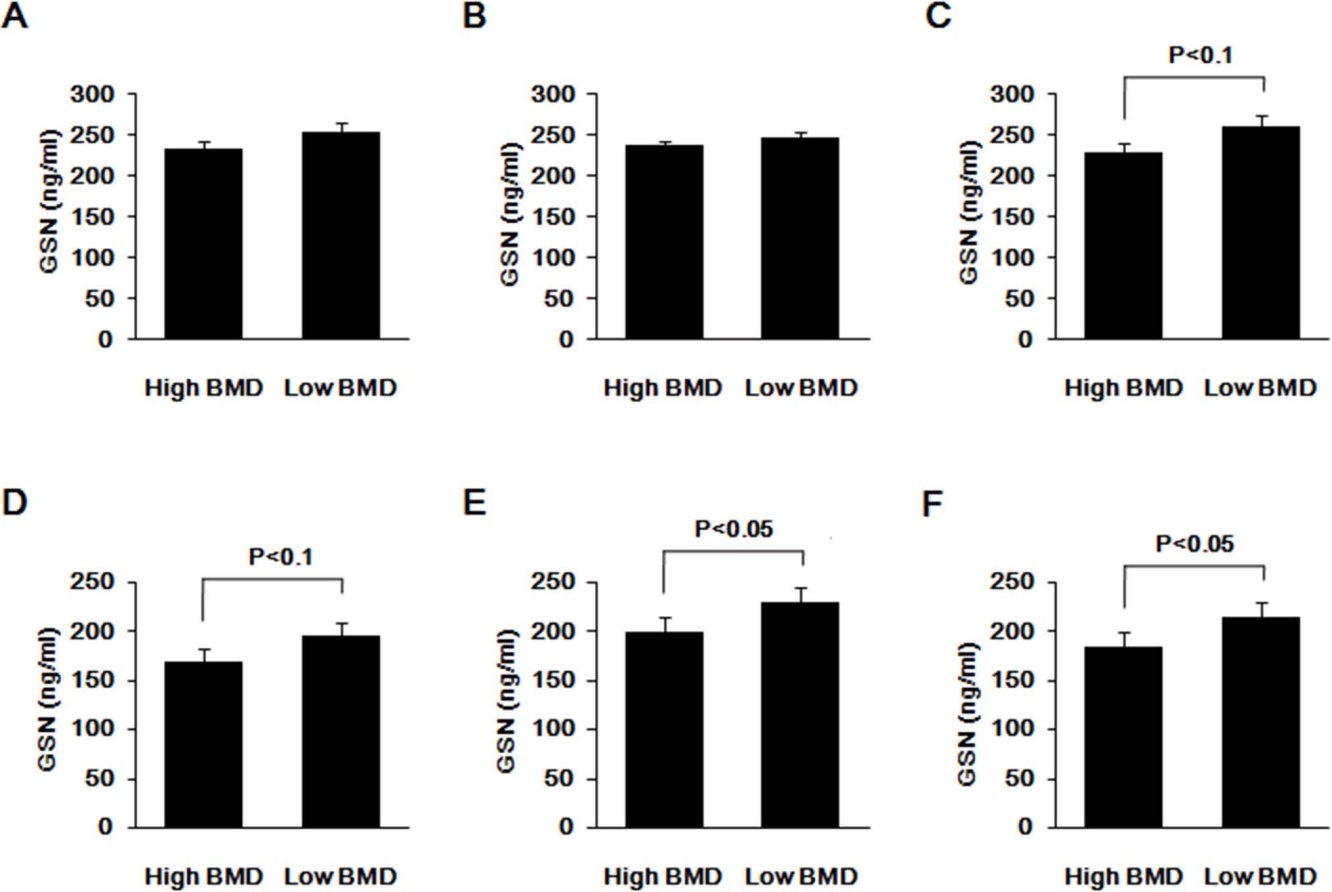
Difference in plasma GSN levels between subjects with extremely high and low BMD. A: Total subjects from the Subgroup1 B: Males from the Subgroup 1 C: Females from the Subgroup 1 D: Females in the Subgroup 2 E: Females in the Subgroup 3 F: Females pooled from the Subgroups 2 and 3. Due to the significant effects of age, BMI and P1NP on BMD, we adopted analysis of covariance to adjust the confounding effects for three Subgroups to compare whether there were statistical differences between high and low subjects. Data was described as mean ± SD.

We tested the difference in plasma GSN level between high vs. low BMD subjects. ELISA assay measured that the plasma GSN protein concentrations ranged from 89.65 to 463.73 ng/ml in the sample. Considering the effect of age, BMI and P1NP on BMD, we adopted analysis of covariance to adjust the confounding effects. In the Subgroup 1 no difference in plasma GSN concentration was observed in the total subjects (P = 0.116) and between the males and females (P = 0.977). Considering that our previous findings on GSN and OP were generated from females[14,15], gender-stratified tests were conducted accordingly in Subgroup 1. Interestingly, we found that the difference in plasma GSN concentration was insignificant in males (P = 0.665), but suggestively significant in females (P = 0.093) (**Fig 2**). Consistent with the findings in the Subgroup 1, the difference was suggestively significant (P = 0.095) in the replication sample Subgroup 2, as well. In the validation sample in Subgroup 3, plasma GSN level was 1.16-fold elevated in the low BMD subjects, when compared to the high BMD subjects (230.08±96.84 vs. 199.16±60.93 ng/ml). The difference was significant (P = 0.037) and much stronger than in the Subgroup 1 or 2. With all females in Subgroups 2 and 3 pooled together, statistical analysis with an increased power showed that the difference in plasma GSN concentration was even more significant in low vs. high BMD subjects (P = 0.016) (**Fig 2**).

### The correlation of plasma GSN with BMD and other indexes

**Fig 3:**
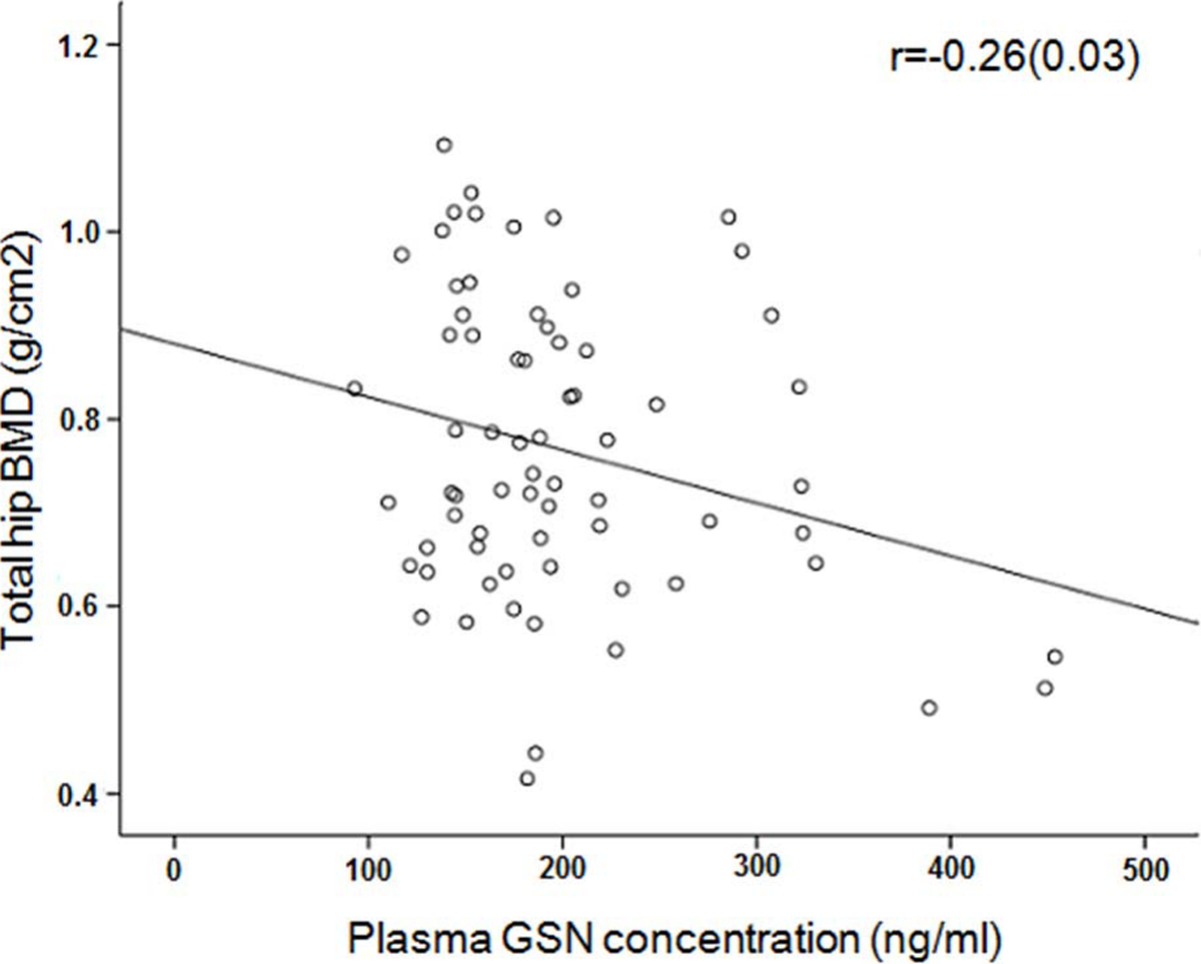
Correlation of plasma GSN concentration with total hip BMD. Partial correlation analysis was conducted to analyze the association of plasma GSN and total hip BMD in the pooled subjects from the Subgroups 2 and 3. We adjusted the effect of covariates BMI, age, P1NP on BMD.

Pearson’s correlation analysis in pooling sample of the Subgroup2 and Subgroup3 showed the suggestive correlation between plasma GSN and BMD (r = -0.19, P = 0.081). Due to the significant correlation of BMD with PINP (r = -0.35, P = 0.002), age (r = -0.38, P<0.001), and BMI (r = 0.70, P<0.001), we adopted partial correlations to eliminate the effect of P1NP, age and BMI on BMD. There was significant negative correlation of GSN with BMD (r = -0.26, P = 0.033) (**Fig 3**). Correlation analyses for GSN with bone turnover markers and metabolic indexes showed that only blood glucose present positive correlation with plasma GSN in the Subgroup 1 and the Male part, but plasma P1NP or β-CTX had no correlation with plasma GSN.

## Discussion

This study represented the first effort investigating the relationship of plasma GSN and BMD at the population level. The results displayed that the plasma GSN levels were significantly up-regulated in extremely low vs. high BMD subjects and negatively correlated with hip BMD in Chinese postmenopausal women.

Extreme sampling adopted in our study is an economic study strategy. It is representative and more powerful to detect some potential biomarkers that have significant differences on the base of small sample sizes over the random-sampling scheme[16,17].Based on a total of 6,308 Chinese subjects, we only utilized 160 subjects to test the association of plasma GSN levels with hip BMD. Since the Subgroup1 and Subgroup 2 were selected from the same Group 1, and the tested numbers of female subject were nearly equal, the differences in plasma GSN levels between the low and high BMD subjects are similar (P = 0.093, 0.095). Comparatively speaking, the Subgroup 3 was generated from the Group 2 (N = 2,486), which was larger than the Group 1 in sample size, hence offered more extreme sampling. It has been recognized that the more extreme sample we selected, the stronger the statistical power we obtained [18]. Coincidently, the difference of plasma GSN level as detected in the Subgroup 3 was more significant than in either Subgroup1 or 2. As expected, with a larger sample size in the pooled sample of Subgroups 2 and 3 selected from a total of 3,585 females (1,099 in Group 1 and 2,486 in Group 2), the difference in plasma GSN level between low vs. high BMD subjects was even more significant. Collectively, the results demonstrated that plasma GSN was associated with hip BMD in old Chinese females.

GSN plays an important role in severing and capping actin filament in actin cytoskeleton organization [6]. It was critical for podosome assembly, rapid cell movements and signal transduction [13]. Podosomes are cytoskeletal structures where a rapid polymerization/depolymerization of actin occurs[19], which are of great importance for cell adhesion, mechanical perception and matrix remodeling in osteoclasts, activated endothelial cells, macrophages and dendritic cells[20–22]. Osteoclast can degrade bone matrix including extracellular matrix and some mineral composition, which are critical for bone remodeling and maintenance of calcium balance [23]. The activation of osteoclast for bone resorption process relies on the integrity of the actin cytoskeleton and adhesion and motility function of podosomes[24]. Previous study showed that GSN deficiency would lead to the inability to produce podosomes, the abnormal actin cytoskeletal architecture; and the reduced rates of osteoclast motility; and blocked podosome-related signal transduction; and eventually cause decreased bone resorption; and increased bone mass and strength[13].

Plasma GSN level presented no significant difference between two genders. However, significant differences in plasma GSN level were observed between high and low BMD subjects in the females specifically. The findings imply that the function of GSN is probably influenced by gender-specific factors, e.g., sex hormones. Previous study suggested that GSN serves as a regulator in regulating androgen-mediated effects on osteoclastogenesis and bone resorption[24]. Whether GSN interact with estrogen to influence bone metabolism and BMD is unknown and has yet to be investigated in the future.

GSN has two protein isoforms, namely intracellular and extracellular isoforms, of which plasma GSN is different from cytoplasmic GSN[25]. GSN can be expressed and potentially be secreted out from CMCs. As evidenced by two independent clinical proteomics studies, GSN expression in CMCs differs significantly in low vs. high BMD subjects in females of different ethnicities. Herein, GSN in plasma was found weakly but significantly correlated with hip BMD. The above findings strengthen the importance of GSN for bone metabolism, though in-depth studies are needed to ascertain the effect of plasma GSN on bone resorption and bone formation.

## Conclusion

In conclusion, by using extreme sampling strategy in large Chinese postmenopausal female population, we discovered, replicated and validated that plasma GSN levels were significantly higher in extremely low BMD subjects than high BMD in Chinese postmenopausal women. We propose GSN to be a potential biomarker involved in the molecular pathogenesis of OP.

## Supporting information

S1 Table The original information of Subgroup 1. (XLSX)

S2 Table The original information of Subgroup 2. (XLSX)

S3 Table The original information of Subgroup 3. (XLSX)
